# Availability and Readability of Emergency Preparedness Materials for Deaf and Hard-of-Hearing and Older Adult Populations: Issues and Assessments

**DOI:** 10.1371/journal.pone.0055614

**Published:** 2013-02-25

**Authors:** Linda Neuhauser, Susan L. Ivey, Debbie Huang, Alina Engelman, Winston Tseng, Donna Dahrouge, Sidhanta Gurung, Melissa Kealey

**Affiliations:** Health Research for Action Center, School of Public Health, University of California, Berkeley, California, United States of America; Federal University of Rio de Janeiro, Brazil

## Abstract

A major public health challenge is to communicate effectively with vulnerable populations about preparing for disasters and other health emergencies. People who are Deaf or Hard of Hearing (Deaf/HH) and older adults are particularly vulnerable during health emergencies and require communications that are accessible and understandable. Although health literacy studies indicate that the readability of health communication materials often exceeds people’s literacy levels, we could find no research about the readability of emergency preparedness materials (EPM) intended for Deaf/HH and older adult populations. The objective of this study was to explore issues related to EPM for Deaf/HH and older adult populations, to assess the availability and readability of materials for these populations, and to recommend improvements. In two California counties, we interviewed staff at 14 community-based organizations (CBOs) serving Deaf/HH clients and 20 CBOs serving older adults selected from a stratified, random sample of 227 CBOs. We collected 40 EPM from 10 CBOs and 2 public health departments and 40 EPM from 14 local and national websites with EPM for the public. We used computerized assessments to test the U.S. grade reading levels of the 16 eligible CBO and health department EPM, and the 18 eligible website materials. Results showed that less than half of CBOs had EPM for their clients. All EPM intended for clients of Deaf/HH-serving CBOs tested above the recommended 4^th^ grade reading level, and 91% of the materials intended for clients of older adult-serving CBOs scored above the recommended 6^th^ grade level. EPM for these populations should be widely available through CBOs and public health departments, adhere to health literacy principles, and be accessible in alternative formats including American Sign Language. Developers should engage the intended users of EPM as co-designers and testers. This study adds to the limited literature about EPM for these populations.

## Introduction: Emergency preparedness communication for vulnerable populations

A major public health challenge is to communicate effectively with diverse and vulnerable audiences about preparing for and responding to disasters and other public health emergencies. During the past 50 years, local community organizations and public health departments in the United States (U.S.) have developed an impressive number of emergency preparedness communications for the public. National health agencies, such as the U.S. Centers for Disease and Prevention (CDC), the U.S. Federal Emergency Management Agency, the U.S. Department of Homeland Security, and many others, have also developed such materials.

A key concern is whether these materials can be accessed, understood and used by the intended audiences. A growing body of research about health literacy indicates that vulnerable populations face many barriers to accessing, understanding and using health information to make important decisions–such as preparing for or responding to emergencies. During the past two decades, researchers have begun examining associations between the needs of diverse populations for emergency communications and the quality of materials available to them. We conducted a search of the scientific literature about these issues (described in the Methods section below) and we summarized key findings from those studies in this section.

Substantial evidence exists that all-hazards emergency preparedness and response efforts are not effectively reaching vulnerable populations in the U.S, [1]–[5] especially those who have barriers related to literacy, language, culture, or disabilities. Over 90 million Americans have low health literacy [6], 22 million have limited English proficiency [7], and over 48 million are Deaf or hard-of-hearing. [8] (In this paper, we use “Deaf/HH” to refer to people who identify as members of the Deaf community and to many other populations with barriers to hearing). These groups face higher risks of injury, death, and property loss as documented in recent U.S. disasters [2], [4], [9]–[11] including the terrorist attacks in the US on September 11, 2001, Hurricane Katrina and Hurricane Rita (in the Gulf Coast area), and the 2007 California wildfires.

For example, at the time of Hurricane Katrina in 2005, only 15% of the population in New Orleans was age 60 or older; but 70%–73% of the deaths attributed to Hurricane Katrina were reported to have occurred among the elderly. [10], [12] Older people also suffered significantly more injuries and death than younger people in the Chicago Heat Wave of 1995. [13], A study of the Saragosa, Texas, tornado [11] documented deaths among Spanish-speaking residents that occurred because warnings were not correctly translated into Spanish. People in the Deaf community had few sources of information and were especially vulnerable during the September 11, 2001 World Trade Center and Pentagon attacks, and during the Hurricane Katrina disaster. [4], [15]

A growing scientific literature documents how the unique needs of these vulnerable groups put them at high risk for lack of preparedness and increased morbidity and mortality during emergencies. [9], [12], [14], [16]–[22] U.S. federal government reports have also documented the critical nature of disparities for vulnerable populations and recommend actions to improve these problems. A 2008 Government Accountability Office report details key gaps in federal and state mass care planning for persons with disabilities, calling this “one of the most serious deficiencies” in current state plans. [2] Likewise, a 2008 Department of Homeland Security report [1] mandates that state and community preparedness plans take into account the needs of individuals with special needs, thus building on the 2004 executive order about emergency preparedness for individuals with disabilities. [23]

Findings from the scientific literature and mandates from federal reports point to serious weaknesses in emergency communication issues for vulnerable populations. As a result, risk communication to high-risk groups has often been ineffective because of barriers related to literacy, language, culture, or disability. [23]

### Health Literacy and Vulnerable Populations

Since the 1990’s, health literacy has emerged as an important communication issue, [24]–[26] including for information about emergency preparedness and response. Health literacy is defined by the US Institute of Medicine [26] as “the degree to which individuals have the capacity to obtain, process, and understand basic health information and services needed to make appropriate health decisions.” The World Health Organization defines health literacy as “the cognitive and social skills and ability of individuals to gain access to, understand and use information in ways which promote and maintain good health.” [27] Health literacy becomes a problem when communication resources and approaches are not well matched with the abilities of the intended recipients.

People’s health literacy abilities are generally measured by standardized tests that assess their skills in understanding written information. In the U.S., the National Assessment of Adult Literacy (NAAL) [6] is the best source of population-based data about Americans’ health literacy. Findings from the NAAL indicate that more than half of the U.S. population has low health literacy and is likely to experience significant communication barriers in using health information, including health-related risk or emergency communications. Only the 12% of the respondents who scored as “proficient” in health literacy were able to answer questions correctly about health literacy tasks routinely required of Americans. A further problem is that during a crisis, even people with high health literacy may find it difficult to understand and act on emergency communication. [28] Results of the International Adult Literacy Survey showed that low heath literacy is a problem in all 25 countries where the survey was conducted. [29], [30] For example, survey findings estimated that 60% of Canadian adults have low health literacy.

In the U.S., low health literacy disproportionately affects vulnerable populations, including older adults, people with disabilities (i.e., specific “access and functional needs” [31]), and ethnic minority groups. [26] Likewise, findings from other countries that have participated in the International Adult Literacy Survey show literacy barriers are more likely among less educated, older, and minority groups and among people with specific access and functional needs. The rapidly growing body of evidence on the impact of low literacy in health care and other areas of functioning leads communication experts to recommend matching text readability closely to audience reading levels. [32] In the U.S., the average adult reading level is estimated to be between the 7^th^ and 9^th^ grade, and an estimated 20% of American adults read at or below the 5^th^ grade. [33], [34] Older adult and Deaf/HH populations face additional literacy challenges that lower their estimated reading levels below the overall U.S. average.

### Health Literacy and Older Adult Populations

Research has shown that people 65 and older have the lowest health literacy skills of any adult age group studied in the NAAL survey. Other studies on older adults corroborate this finding and suggest a relationship to cognitive decline and other factors related to aging. [6], [35]–[37] For these reasons, health communication scholars and practitioners generally recommend that communication materials for this population be written at a 6^th^ grade level or lower. [38]–[40] In addition, medical conditions that are more common with aging, such as stroke, and vision and hearing impairments, affect older adults’ abilities to access and use emergency preparedness communications. For example in the U.S., hearing loss affects one in three people older than 60, and half of people older than 85. [41] Further, in keeping with the principles of Adult Learning Theory, [42] older adults also prefer communication that is more contextual, builds on their prior knowledge, and is oriented to solving problems–rather than abstract or didactic recommendations. These principles take into consideration the significant life experience and knowledge of older adults–an important factor related to health literacy.

### Health Literacy and Deaf and Hard-of-hearing Populations

Although Deaf/HH sub-groups were not specifically studied in the NAAL, health literacy is a major problem for this population. The estimated 48 million [8] people in the U.S. in this population comprise many sub-populations, including those who are late-deafened, deaf-blind, hard-of-hearing, and oral deaf people whose native language is spoken English and choose to have a hearing, rather than Deaf, cultural orientation. [43] As noted above, a significant proportion of older adults become hard-of-hearing or deaf. Deaf/HH sub-populations use a variety of communication modalities including American Sign Language (ASL), Signed Exact English (SEE), Pidgin Signed English (PSE), Cued Speech, lip-reading and spoken English, and have varying degrees of English and/or other language literacy skills. Many misunderstandings exist about the health literacy capacities and communication preferences of Deaf/HH populations, especially about ASL users.

ASL: Researchers, practitioners and policy-makers often incorrectly categorize Deaf people who use ASL with other “limited-English proficiency” (LEP) groups and recommend that English or other language materials be “translated” into ASL. However, this greatly underestimates the challenge of adapting information for ASL-users. One fundamental issue is that many people who use ASL as their primary mode of communication also share a unique set of values, social behaviors and other characteristics recognized as “Deaf culture.” [44] Such cultural features are often overlooked in adapting communication resources for ASL users. ASL is a “visual, gestural language” that differs greatly from English and other verbal languages. [45]

An important literacy consideration is that ASL differs from English in its syntax, grammar and idiom vocabulary. [46] Words in English or other languages may not exist in ASL, as has been found for some emergency preparedness terms. It is estimated that 90% of Deaf children are born to hearing parents [47], who are often not able to provide their children with a strong language foundation for effective communication. According to Barnett and colleagues [48], "many adults deaf since birth or early childhood have low health literacy…[and] deaf adult sign language users’ knowledge of English medical terminology is similar to that of non-English–speaking immigrants to the United States." In one survey, Deaf respondents who were tested on health-related vocabulary in English did poorly despite being well educated. [49], [50] In addition, because Deaf people have significant barriers to receiving information from sources commonly used by hearing people such as TV (including lack of, or inconsistent use of captioning/subtitles), radio, educational presentations, etc., they may have significant “fund of information”–or factual–limitations relative to people who can access hearing media. [49]

There is a wide variation among ASL users in their English literacy abilities. Although research is limited, LaVigne and Vernon [51] estimate that 30% of Deaf adults have both weak ASL skills and also read below a 3^rd^ grade level, and 60% use ASL effectively, but read between the 3^rd^–6^th^ grade level. They estimate that only about 10% of the Deaf population who earn college degrees are both fluent ASL users and fluent readers. The only available estimate of English literacy for the deaf population comes from a study of deaf high school seniors who were found to read between the 3^rd^ and 4^th^ grade levels. [52]–[54] Based on this finding, communication scholars and practitioners generally recommend that communication for Deaf populations be written at the 4^th^ grade level or below. Experts also recommend that, given the literacy constraints and communication preferences of Deaf populations, communication resources also be developed in ASL with the close participation of the Deaf/HH users. [49]

### Readability of Emergency Preparedness Communications

Because Deaf/HH and older adult populations suffer disproportionate rates of morbidity and mortality during disasters and also have low health literacy capacities, it is thought that a major problem may be that they do not receive emergency preparedness communication adapted to their literacy levels. In addition, an extensive body of literature–over 800 studies conducted during the past two decades–shows that most health information is written at levels that exceed people’s abilities to understand it. [30], [55] However, surprisingly few studies have assessed the readability of emergency preparedness materials–for any population. This is an especially critical gap, because risk communication places additional literacy burdens on readers. Research has indicated that the emotionally laden crisis content of risk communication makes it significantly harder for people to comprehend. [56]

Friedman and colleagues [57] evaluated the readability of online disaster and emergency preparedness materials on 50 websites that provided such information for the public. They found a mean readability score of grade 10.7 using the Flesch-Kinkaid test, and the mean score using the Flesch Reading Ease test was “difficult to read” (12^th^ grade or above). Although some of these websites designated material for specific age groups, including older adults, the study did not specifically analyze the readability of materials for older adults. The study’s general findings are similar to results of readability studies of general online health content. [33] Zarcadoolas and colleagues [58] conducted research on a postcard about anthrax that was mailed to U.S. households in 2001 and found that it had a reading level significantly higher than that of the general population. Another study found that the mean reading level (Flesch-Kinkaid test) of a sample of preparedness communication on emergency management websites in the state of Maryland (U.S.) was grade 9.45. [59] We could find no research about the readability of emergency preparedness communication (print or online) intended specifically for Deaf/HH or older adult populations.

### Suitability of Emergency Preparedness Materials

Besides readability, other factors affect the reading ease and usability of health, risk, and emergency preparedness communication for diverse audiences, especially those with low literacy capacities. Such characteristics are often grouped into “clear communication” or “plain language” design criteria. There is no agreed upon single set of such criteria, but they are described in U.S. government reports and guides, including the Department of Health and Human Services’ *Quick Guide to Health Literacy* and its *Toolkit for Making Written Material Clear and Effective*, and the Centers for Disease Control and Prevention’s *Simply Put*. [39], [60], [61] Key design criteria are also embodied in tools, such as the Suitability Assessment of Materials (SAM), used to assess health communication materials. The SAM is the most validated and most commonly used tool and includes readability and 21 other evidence-based design principles as shown in Table 1. [58], [62], [63] We could find only one study that assessed SAM factors for emergency preparedness communication. In the previously mentioned study, Friedman and colleagues [57] used the SAM tool to assess emergency preparedness content on 50 websites. They found a mean SAM score of 48%, meaning that the website material was “below average suitability.”

**Table 1:** Suitability Assessment of Materials (SAM)*.

Factor to be Rated	Score	Comments
	0 = Not Suitable1 = Adequate2 = Superior	
**1. Content**		
a. Purpose is evident		
b. Content about behaviors		
c. Scope is limited		
d. Summary or review included		
**2. Literacy Demand**		
a. Reading grade level		
b. Writing style, active voice		
c. Vocabulary with common words		
d. Context given first		
e. Learning aids via “road signs”		
**3. Graphics**		
a. Cover graphic showing purpose		
b. Type of graphics		
c. Relevance of illustrations		
d. List, tables, etc. explained		
e. Captions used for graphics		
**4. Layout and Typography**		
a. Layout easy to follow		
b. Typography appropriate		
c. Subheads (“chunking”) used		
**5. Learning Stimulation and Motivation**		
a. Interaction used		
b. Behaviors modeled and specific		
c. Motivation/self-efficacy		
**6. Cultural Appropriateness**		
a. Match in logic, language, experience		
b. Cultural image and examples		
**Total SAM Score:**		
**Total Possible Score:**		
**Percent Score: % Not Suitable Material**		

Interpretation of SAM percentage ratings:

70–100% = superior material.

40–69% = adequate material.

0–39% = not suitable material.

* The SAM tool was validated with 172 health care providers from several cultures, including Southwest Asians, Native Americans, and African Americans, as well as students and faculty from the University of North Carolina School of public Health and Johns Hopkins School of Medicine. The SAM was developed under the Johns Hopkins School of Medicine project, “Nutrition Education in Urban African Americans,” funded by the National Institutes of Health, National Heart, Lung and Blood Institute, Bethesda, MD, 1993.

### Availability of Emergency Preparedness Materials in Community Settings

Health and risk communication experts emphasize that information resources should not only be comprehensible, but also available and delivered by trusted sources–especially for vulnerable populations. [18], [56], [64]–[68] Substantial research now indicates that preparedness communication for vulnerable populations will only be successful if it is moved beyond the individual to community systems. [17], [68], [69] Community-based and other grassroots organizations are key players in this approach and have an important role in preparedness communication for vulnerable groups. [70] Although we mentioned earlier that several studies exist about the readability of online emergency preparedness materials, we found no studies of the availability of emergency preparedness materials *distributed* to the public in a geographic area.

### Gaps in the Literature

Our review of the literature found ample research about the disproportionate vulnerability of Deaf/HH, older adult and other populations during and after health emergencies and disasters. Research findings have identified important risk factors for the vulnerability of these populations: their notably lower health literacy capacities, and the lack of accessible and comprehensible information for them. With the exception of one national postcard study, [58] research about the readability of emergency preparedness materials is limited to web-based materials that may not be accessible to populations with significant literacy barriers. Our study was intended to help fill gaps in the literature by assessing the availability and readability of printed emergency preparedness materials provided by community organizations to Deaf/HH and older adult populations.

### Study Objectives

The U.S. Centers for Disease Control and Prevention (CDC) has funded a national network of Preparedness and Emergency Response Research Centers and supported our center at the University of California, Berkeley to conduct a study of emergency communication for vulnerable populations.

The purpose of this study was to 1) review the literature related to emergency preparedness communications for vulnerable populations, especially for Deaf/HH and older adult populations, 2) explore the availability and readability of printed emergency preparedness materials from community-based organizations serving Deaf/HH and older adult populations in the San Francisco Bay Area (California), 3) examine the availability and readability of web-based emergency preparedness resources for these populations from key local and national websites that include emergency preparedness information, and 4) recommend readability benchmarks and processes to improve emergency preparedness communication for these populations.

## Methods

### Protection of Human Subjects

**Figure 1:**
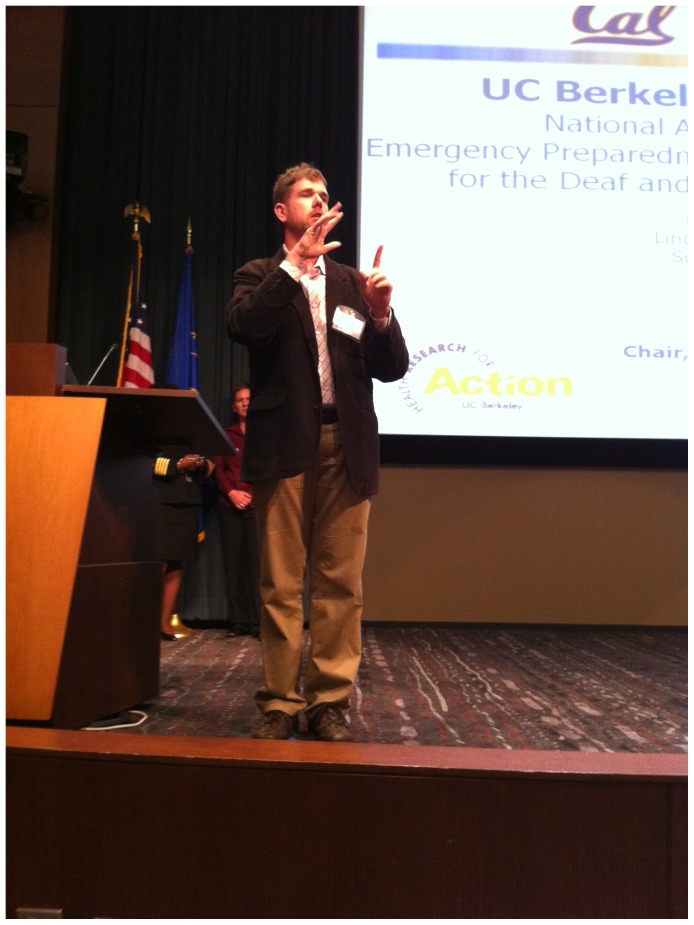
James R. Brune, Executive Director, Deaf Counseling, Advocacy and Referral Agency; PERRC National Advisory Board member presents at Preparedness and Emergency Response Research Center meeting at Centers for Disease Control and Prevention.

All protocols were reviewed by the institutional review board at the University of California, Berkeley and found to be exempt. The individual in Figure 1 gave written informed consent for PLOS ONE to publish his image.

### Literature Review

We searched *Pubmed* and *Web of Science* databases for published studies to date that included multiple combinations of the following key word search terms: seniors, literacy, clear communication, disaster communication, emergency preparedness communication, health communication, health literacy, plain language, preparedness communication, risk communication, communication, comprehension, deaf, disability, disabled, disaster, disaster preparedness, earthquake, emergency preparedness, flood, hurricane, older adults, preparedness, readability, special needs, tornado, and vulnerable population. The search yielded a total of 7,233 publications. We reviewed abstracts of those publications and selected a total of 78 relevant publications for full review. We included relevant findings and citations from those publications in this paper.

### Advisory Boards

In keeping with the principles of Community-Based Participatory Research [71] and as part of our larger CDC-funded research project about emergency preparedness communication for vulnerable populations, we established two advisory boards that helped guide this study. Beginning in 2009, we convened a “National Advisory Board” of key experts from the Deaf community and others involved with these issues in the U.S. to help us develop the study design, interpret study results, and create national recommendations about improving emergency communication for Deaf/HH populations. James R. Brune, chair of the National Advisory Board and executive director of the Deaf Counseling, Advocacy and Referral Agency is shown in Figure 1 at a CDC meeting. In the same year, we also established a local “Community Advisory Board” comprised of experts, providers and advocates in the San Francisco Bay Area (California) who served Deaf/HH or older adult populations and who were involved with emergency preparedness communication issues. This community board has met since 2009 and helped our research team develop the local study design, make connections with community-based organizations, interpret results, and produce recommendations.

### Sampling

We collected printed emergency preparedness materials from staff at local community-based organizations (CBOs) serving Deaf/HH and older adult clients. We also selected materials from local and national organizations that provided emergency preparedness materials on their websites.

#### Sample of Deaf/HH-serving CBOs

Because there were a limited number of Deaf/HH-serving CBOs in the San Francisco Bay Area, we developed a database of 20 such organizations that included the vast majority of such CBOs located in two San Francisco Bay Area counties.

#### Sample of older adult-serving CBOs

In contrast, because of the very large number of CBOs serving older adults in this area, we limited our sampling area to just one county – Alameda County – to obtain an equivalent total sample of 20 CBOs serving this population. As described by Kealey and colleagues [72], we developed a stratified sampling frame of 227 CBOs comprised of four strata of organizations that served non-institutionalized older adults: senior centers, senior residences, in-home services, and community health organizations that offer multiple services. From this sampling frame, we then randomly selected five CBOs from each of the four organizational categories for a total study sample of 20 CBOs. If an organization in the CBOs sample did not agree to participate, we randomly selected a replacement organization from the same stratum until we reached the quota of 5 participating organizations in each of the 4 strata.

#### Sample of public health departments

We also selected two local health departments to include in the overall CBO sample.

#### Collection of materials from local CBOs

We interviewed a key informant (KI) at each of the CBOs in the two samples. A research team member fluent in American Sign Language (ASL) interviewed KIs who were Deaf. Using a semi-structured questionnaire, we asked each KI about whether the CBO provided printed emergency preparedness materials to its clients, and if so, to provide us with those materials. We also asked KIs about issues and recommendations related to emergency preparedness materials for their clients. From the collected materials, we excluded emergency preparedness materials that were not intended for clients of each CBO. For example, we specifically excluded materials that were about facility preparedness, such as internal administrative plans for evacuation or other safety procedures.

#### Selection of website materials from local and national organizations

We developed a list of key local and national organizations (such as the U.S. Centers for Disease Control and Prevention (CDC), the U.S. Federal Emergency Management Agency, The American Red Cross and local public health departments) that provide emergency preparedness materials for the public on their websites. From this list, we searched each organization’s website to identify emergency preparedness materials specifically designated for Deaf/HH and older adult populations. Because we found few materials designated for these populations, we then selected an additional convenience sample of materials intended for the general public to reach a quota of 40 national materials for testing–to match the number of the 40 materials collected from the KIs at the Deaf/HH- and older adult-serving CBOs who participated in the interviews.

### Availability Assessment of Materials for CBO Clients and from Websites

We assessed the availability of emergency preparedness materials from CBOs serving Deaf/HH and older adult clients both from the responses of the KIs about the availability of these materials for clients, and also by collecting materials from the CBOs. We also noted the general availability of emergency preparedness materials contained in the local and national websites in our sample.

### Readability Assessment of Materials

#### Selection of materials for testing

To ensure compatibility with the readability software used, we limited client materials in the final sample to those that were written in English, and had at least 100 consecutive words to test–as required by the software’s algorithm, and were non-duplicative of other materials collected.

#### Readability testing

We conducted readability testing on the selected materials using Readability Plus software. [73] Readability testing is a standardized method to estimate the US grade reading level of text content. Because the reliability of readability test scores differs at various reading levels–as do their underlying readability formulas–we tested materials using three widely used and standardized readability tests included in the software: Flesch Reading Ease Scale [74] Fry Graph [38], and SMOG. [75] We excluded use of the popular Flesch-Kinkaid test that is incorporated into Microsoft Word and that has been used in past assessments of online emergency preparedness content [59] because its underlying formula is truncated at the 12^th^ grade level, and the test frequently presents falsely low evaluations. [76] We then created a table that showed the reading levels scores of each of the three readability tests for each material tested.

#### Readability benchmarks

we set maximum reading level benchmarks based on available research about the literacy levels of Deaf/HH and older adult populations and recommendations from communication experts, mentioned above. [77]–[79] The maximum reading level benchmark for Deaf/HH populations was set at the 4^th^ grade level, and at the 6^th^ grade level for older adult populations.

### Suitability Assessment of Materials

Although this study was focused on readability and availability of emergency preparedness materials, we wanted to illustrate the process of assessing additional design factors thought to be important for “plain language communication.” We selected the Suitability Assessment of Materials (SAM) [62], [63] tool (described in the Introduction) to assess such design criteria. Table 1 shows the 22 communication elements included in this test, such as text density, list lengths, font size, graphics, and cultural relevance.

We selected three materials to test using the SAM. We selected an H1N1 information sheet from a local CBO serving older adults. We chose this resource because it had tested at the recommended readability level for older adults and we wanted to see if other design factors would also test well. From a Deaf/HH-serving CBO, we selected a resource about emergency evacuation for children because we thought this topic would be important to this population. Finally, from a national website, we selected a resource about earthquake preparedness because this topic is essential in California. Two members of the research team independently conducted the SAM test on each of the materials. If researchers’ initial scores differed on any item, they conferred until reaching total agreement on that score.

## Results

### CBO Participation and Materials Collected

We conducted a total of 36 interviews: 14 with KIs at CBOs serving Deaf/HH clients, 20 with KIs at CBOs serving older adult clients, and 2 with KIs at health departments. KIs from 6 of the 14 participating Deaf/HH-serving CBOs, and from 4 of 20 CBOs serving older adults, provided copies of their emergency preparedness materials to our study staff for analysis. In addition, KIs from the two public health departments provided such materials to our staff.

KIs from 18 of the 34 (53%) participating CBOs serving Deaf/HH and older adult clients, reported that they did not provide emergency preparedness materials to their clients, and KIs from another 8 Deaf/HH- and older adult-serving CBOs (24%) reported that either they no longer had emergency preparedness materials for clients, could not locate them, or could not provide copies of them to our study staff for some other reason. In summary, copies of emergency preparedness materials for analysis were available from only about 23% of the CBOs serving Deaf/HH and older adult clients. Materials were available from both public health departments.

We collected a total of 40 materials from the 10 CBOs and the 2 public health departments that provided materials. Preparedness topics covered include earthquake preparedness, evacuation preparedness, H1N1 influenza pandemic preparedness, and general emergency preparedness. Many of the materials were duplicative and most were provided to the CBOs from a small number of organizations with expertise in emergency preparedness, such as the American Red Cross, Citizen Emergency Response Teams, Collaborating Agencies Responding to Disasters (a local emergency preparedness training organization), and the local public health and fire departments. As reported by Kealey and colleagues [72], CBOs distributed most of the materials to clients at training sessions or presentations, and/or made them available as flyers for pick-up. Deaf-serving CBOs were more likely to post materials online compared with CBOs serving older adults. Half of the KIs at Deaf/HH-serving CBOs reported having video/DVD materials with captioning, and about 17% reported having materials with video/DVD and ASL interpreting.

KIs reported that major barriers in obtaining emergency preparedness materials were lack of funds, staff time and capacity to create accessible materials. [72] KIs from most Deaf/HH- and older adult-serving CBOs reported that many of their clients did not use computers. Most of the KIs reported that their CBO would benefit from having more emergency preparedness materials to offer clients. They recommended that these materials be simplified and written in “plain language,” available in languages other than English (including ASL), and available in alternative formats for people with various access and functional needs.

### Materials Collected from Websites

We examined the 14 local and national websites for emergency preparedness materials specifically aimed at Deaf/HH (3 found) and older adult populations (14 found), and then selected an additional 23 materials intended for the general public to yield the desired total of 40 materials. Although we found few materials specifically intended for our focal populations, most of the websites contained ample all-hazards emergency preparedness materials for the general public.

### Readability Testing of Local CBO and Public Health Materials

Of the 40 materials collected from the 12 local CBOs and public health departments, we found 16 that met the criteria of relevance for client populations, were compatible with the readability software and were not duplicative of other local materials collected. We tested those materials and results are shown in Table 2. Results showed that all of the materials provided by Deaf/HH-serving CBOs exceeded the recommended maximum 4^th^ grade reading level, and half of them tested in the 10^th^ grade to college level range. Of materials tested from CBOs serving older adults, only one (“H1N1 Influenza Questions and Answers”) tested at or below the maximum 6^th^ grade reading level. Likewise, materials from public health departments all tested above the 6^th^ grade level–exceeding the maximum level for both target populations.

**Table 2:** Readability Scores* by Readability Test for Materials Provided by Local Community-based Organizations and Public Health Departments.

Source of Materials: (CBOor public health)	Material Name	SMOG	Flesch Reading Ease	Fry	Overall Range
Deaf/HH	Safe Schools: A Planning Guide for Action	11.3	10–12	14	10–14
Deaf/HH	Earthquake Preparedness Tips from the California Governor’sOffice of Emergency Services	7.8	7	7	7–8
Deaf/HH	Earthquake Preparedness	12.1	10–12	10	10-College
Deaf/HH	Letter to Parents/Guardians 8-20-2010	11.8	10–12	11	10–12
Deaf/HH	CEID and Sunshine Emergency ID Card	10.8	8–9th	8	8–11
Deaf/HH	CEID Disaster Plan	14.9	College	16	College
Deaf/HH	The Seven Steps to Earthquake Safety	11.2	10–12	12	10–12
Deaf/HH	Emergency Checklist	9.3	7	n/a^1^	7–9
Deaf/HH	http://72hours.org/under “www.sfgov.org”	10.4	8–9	8	8–10
Deaf/HH	Actions for Emergency Preparedness	14.2	College	n/a^1^	College
Older Adults	CDC Says “Take 3” Steps to Fight the Flu	10.6	8–9	9	8–11
Older Adults	H1N1 Influenza Questions and Answers	6.2	5	3	3–6
Older Adults	Personal Preparedness: Seniors	8.8	7	7	7–9
Public Health	Emergency Preparedness, 5 Critical Steps: To Prepare YourFamily for an Emergency	11.5	10–12	n/a^1^	10–12
Public Health	WHACK Prevention Messages - Teacher Activities	11.9	10–12	11	10–12
Public Health	Your Guide to Preparing for Pandemic Flu	10	8–9	8	8–10

* Scores estimate US grade reading level of the tested material.

1 The material was outside the Fry Graph’s range.

### Readability Testing of Materials from Websites

Of the 40 materials collected from 14 local and national websites that provide emergency preparedness materials, 18 met the testing criteria (analyzable by the readability software, and not duplicative of other website materials collected). The results of those readability tests are shown in Table 3. Of the website materials tested, only one was specifically aimed at Deaf/HH populations; that resource scored between the 10^th^ and 12^th^ grade reading levels–far exceeding the recommended maximum 4^th^ grade level. Of materials intended for older adults, only one resource (“Earthquake Tips for Seniors”) tested at 6^th^ grade level for 1 of the tests, and at 7^th^ and 9^th^ grade for the other 2 tests, respectively. Of other website materials that were not specifically designated for either of the target populations, all tested above the 6^th^ grade level and most tested at the 10^th^ grade to college reading levels.

**Table 3:** Readability Scores* by Readability Test for Materials Collected from Websites of Local and National Organizations.

IntendedAudience	Material Name	SMOG	Flesch Reading Ease	Fry	Overall Range
Deaf/HH	Tips for People with Hearing Impairments	12	10–12th	n/a^1^	10–12
Older Adults	Dealing with Disaster	10.2	8–9th	10	8–10.2
Older Adults	Earthquake Safety Tips for Seniors	8.95	7th	6	6–9
Older Adults	Emergency Evacuation Preparedness – A Guide for People with Disabilities and Other Activity Limitations	14.6	College	12–13	12-College
Older Adults	Preparedness Fact Sheets - H1N1	8.45	7th	7.5	7–9
Older Adults	Step 1: Get a kit: Gather Emergency Supplies	11.15	10–12th	11	10–12
Older Adults	Step 2: Make a plan: Develop a Family Disaster Plan	12.15	10–12th	11.5	10-College
General	How to Develop a Disaster Action Plan for Older, Distant Relatives	14.05	College	13	College
General	Emergency Food Supply	12	10–12th	9	9–12
General	Sewage Disposal in an Emergency	12.5	College	13.5–14	College
General	Heat Wave: Are you Prepared?	10.8	10–12th	15	10-College
General	Your Guide to Preparing for Pandemic Flu	10.5	8–9th	9–9.5	8–11
General	5 Steps to Safety	10.55	8–9th	8	8–11
General	Are you Ready? An In-depth Guide to Citizen Preparedness	12.9	10–12th	12.5	10-College
General	People with Disabilities and Other Access and Functional Needs – Preparing and Planning	13.1	College	14–15	College
General	Emergency Preparedness Checklist	10.65	8–9th	10–11	8–11
General	Preparedness Fact Sheets – Earthquakes	11.3	8–9th	10	8–11
General	Personal Preparedness: Parents and Caregivers	13.4	10–12th	11.5	10-College

* Scores estimate US grade reading level of the tested material.

1 The material was outside the Fry Graph’s range.

### Suitability Assessment of Materials

As shown in Table 4, the “H1N1 Influenza Questions and Answers – the only resource not to exceed the 6^th^ grade reading level for older adult audiences–scored at the “superior” level on SAM criteria. The other two materials tested scored in the “adequate range” (40–69% as indicated in the SAM matrix in Table 1). Overall, SAM results showed that sampled materials had deficiencies in many design criteria such as the lack of adequate graphics, layout, typography and content required for easy-to-use communications.

**Table 4:** Suitability Assessment of Materials Scores for Three Emergency Preparedness Materials.

Material	Intended Audience	Origin of Material	Highest Scoring Area	Lowest Scoring Area	Score
SAM-1 H1N1	Older Adults	Local CBO servingolder adults	Content; Readability; Learning Stimulationand Motivation; Cultural Appropriateness	Graphics; Layout andTypography	33/42 (79%)
SAM-2 Emergencyevacuation information	Deaf/HH	Local CBO servingDeaf/HH	Literacy Demand; Learning Stimulationand Motivation	Content; Graphics; Layoutand Typography	15/28 (54%)
SAM-3 Earthquake preparedness fact sheet	General population	National levelorganization website	Content; Learning and Stimulation;Cultural Appropriateness	Learning Stimulation andMotivation; Graphics;Readability	29/42 (69%)

## Discussion

Substantial evidence shows that vulnerable populations, including Deaf/HH and older adult populations, have low levels of emergency preparedness and experience disproportionately high risks of death and injury during and after emergencies. Research also shows that Deaf/HH and older adult populations have notably lower levels of literacy than the general U.S. population in which adults are estimated to read between the 7^th^ and 9^th^ grade, on average. Although research about the health literacy capacities of Deaf/HH and older adults is limited, current recommendations advise that the readability of health communications not exceed the 4^th^ grade for Deaf/HH populations, and 6^th^ grade for older adults. However, over 800 studies document that the readability of most health and risk information is at the 10^th^ grade or above. Taken together, such findings have prompted concerns about whether Deaf/HH, older adult and other vulnerable populations have adequate access to understandable emergency preparedness information.

In our review of the published literature, we found little information about the readability of emergency preparedness materials for vulnerable populations. Only one study assessed the readability of printed materials (post card about anthrax) and showed that the information notably exceeded average adult reading level. [58] Other research has been limited to several studies of online materials [57], [59] that tested at around the 10^th^ grade level. These few studies about the readability of emergency preparedness materials show results similar to those found in research about general health and risk communication.

We could find no studies about the availability of non-Web-based emergency preparedness communication for Deaf/HH, older adult, or other vulnerable populations. This is important because the literature suggests that these populations often face barriers to accessing and/or understanding online information and may be better served by receiving emergency preparedness information from trusted local community-based organizations. To help fill the research gap, we examined the availability and readability of printed emergency preparedness materials for Deaf/HH and older adult populations in the San Francisco Bay area. Guidance from our National Advisory Board, Community Advisory Board and from many other participants in this study proved invaluable to design, implement and interpret this study.

We found that although about half of community-based organizations (CBOs) reported that they had materials available for these populations, less than a quarter of these organizations could actually provide us with these materials. From our interviews with staff at these organizations, it appeared that the half of CBOs that provided such materials to their clients did so episodically–for example, after a training or presentation. Staff at the CBOs said they would like to have emergency preparedness materials for their clients and especially wanted them to be written in “plain language” and in accessible formats. Staff was rightly concerned about the comprehensibility of the materials they provided. Our research showed that all materials intended for clients of Deaf/HH-serving organizations exceeded the recommended 4^th^ grade reading level, and all but one resource intended for clients of organizations serving older adults exceeded the recommended 6^th^ grade level. Half of the print materials tested in the 10^th^ grade to college range. Our readability testing for web-based materials found similar results. Only one resource intended for older adults tested at the 6^th^ grade level, and most materials tested at the 10^th^ grade to college levels.

In recent years, there has been a rapid increase in the number of emergency preparedness videos in ASL and we noted that some of the CBOs in our study had such resources available for their clients. There are currently no accepted criteria for assessing the comprehensibility of ASL video communications about emergency preparedness. In a separate study, our research team is conducting focus groups with ASL users to explore their understanding of and recommendations related to ASL emergency preparedness videos.

### Improving Emergency Preparedness Materials

Clearly, emergency preparedness communication needs to be improved not only for populations that experience high communication barriers, but also for all populations. Fortunately, there is extensive general evidence-based guidance about developing and testing health and risk communication materials for diverse audiences, including for people with limited health literacy skills. Key resources, previously mentioned in the Introduction, include: [39], [60], [61], [63]

This guidance emphasizes the value of applying known criteria for easier-to-understand communication, such as the 22 factors embodied in the SAM tool. It also recommends that the intended users be closely involved in designing and testing materials. Although user-designed communication is not yet common in developing health and risk communication, there are now a number of helpful models. [49], [53], [80] Suggested processes include: identifying the intended audiences and relevant stakeholders, assessing literacy levels and other communication factors related to the intended users, adhering to known design criteria when developing a first draft of the material, iteratively testing and revising drafts with intended users, developing implementation plans with intended users and stakeholders, and finally, evaluating the effects of the materials. [80]

Participatory design processes are particularly useful to address the complexity of emergency preparedness communication for Deaf/HH, older adult, and other vulnerable populations. As we commented earlier, Deaf/HH populations comprise diverse sub-groups whose communication needs may differ. For example, because Deaf children typically have hearing parents, communication should consider the needs of both parts of the family dyad. Sub-groups of older adults are likewise very diverse, and in some cases materials also need to be useful to caregivers. Matching readability of materials more closely to the literacy levels of the users, and adhering to other known design principles can significantly improve the comprehensibility and usability of resources for these populations. We note, for example, that the U.S. Centers for Disease Control and Prevention (CDC) has made good progress in improving the readability and design of its emergency communications for the public, especially by reducing readability of many materials to the 8^th^ grade level, and some to the 6^th^ grade level. [53] The CDC also demonstrated its commitment to examine barriers and improve communication for Deaf/HH populations by inviting our study team and National Advisory Board members to participate at a CDC meeting about emergency preparedness research. Figure 1 shows Board Chair James R. Brune presenting project findings. Readability and other design factors are helpful guides to develop more comprehensible and usable emergency preparedness materials. However, other factors are also important to ensure that materials are also motivating and actionable within a user’s social context. Because “motivation” and “actionability” cannot be codified as well as readability and other design factors, participatory design with the users is the only way to address these essential aspects of communication. For example, one of our key community partners, Collaborating Agencies Responding to Disasters, advocates that an empowering, positive, and practical action approach to preparedness communication is much more effective than traditional “fear-based” communications. User-centered design is essential to transform communications content in this way.

In addition, the mode of communication can be just as important as the quality of the content. As we have learned from reviewing the literature, from interviewing CBO staff and from the rich input of members of our advisory boards, communication modalities are especially important for users of ASL whose language(s) are not directly translatable into English. Effective health communications will only be created if ASL users or other diverse Deaf persons are co-designers and testers as is being done in some excellent work at the University of Rochester (New York) in the U.S. [81] As this work progresses, more specific recommendations will be available to emergency planners, community organizations and others that serve Deaf/HH groups.

Finally, we have learned from our work with “vulnerable” populations like Deaf/HH and older adults, that it is often these people who are on the cutting edge of communication approaches that can be used to help *all* populations. This reflects the important principle of “universal design” in which designing for those with the most barriers, results in designs that are often better for all. For example, Deaf/HH populations were early champions of e-mail, and now of mobile videophones–both of which have become central to emergency preparedness and response communication. In addition, Deaf/HH groups often form geographic social networks that can share information rapidly among a large number of people in case of emergencies. These kinds of networks may be the key to preparedness for all populations.

### Strengths and Limitations

Our study has a number of limitations. Although we did an extensive search of the literature that yielded over 7,000 publications to review, there may be other relevant research that is not in English, not yet published, or for some other reason not included in our search results. However, the studies cited here likely give a good sense of available research about the readability and availability of emergency preparedness communication for our focal populations.

Our study design had differences in the sampling frame used to select CBOs that served Deaf/HH vs. older adult populations. The number of Deaf/HH-serving organizations in our geographical area was small, so we were able to include all of them in the original sampling frame and interview two-thirds of them. However, the number of older adult-serving organization in the county was quite large, and required a different sampling strategy. We avoided obvious selection bias by grouping older adult organizations into four strata based on shared characteristics and by randomly selecting a sample of 5 organizations from each of the strata. If an organization did not agree to participate or was unreachable, an alternate organization was randomly selected from the same stratum.

We met our goal of interviewing staff at a total of 20 older adult-serving CBOs in our stratified sample, and were able to interview staff at 14 of 20 CBOs that served Deaf/HH clients. However, our overall sample of CBOs was small, and a larger sample might have shown different results. Because we found very low availability of emergency preparedness materials, we doubt that a larger sample would change our findings about the availability of emergency preparedness materials for clients. Likewise, because the readability of all but one material collected from the CBOs was above the recommended reading level for the focal client group, we doubt that a larger sample of materials drawn from CBOs would yield significantly different results. We also noted that because very few CBOs developed their own materials and tended to rely on materials originating from a limited number of organizations, like the American Red Cross or local health departments, we would not predict that results would differ in a larger CBO sample, given that materials would likely come from the same sources.

Our study of the readability of emergency preparedness materials from local and national websites was intended mainly to search for materials designated specifically for Deaf/HH and older adult populations. Because we could find few such materials, we selected an additional convenience sample of materials from websites intended for general populations. These general population materials were not randomly selected, and we cannot generalize the results to represent readability on U.S. websites providing emergency preparedness materials. However our readability results were quite similar to those found in an extensive study of emergency preparedness materials from 50 U.S. websites. [57]

Another limitation relates to readability testing. Because the readability software required at least 100 words of continuous text to assess, not all materials could be tested. Finally, readability software estimates reading level of text–which is a critical measure of utility to users. However, it does not measure clarity of writing, design and layout, credibility, cultural, or linguistic factors, which can also affect how well people can use these materials. We recommend including SAM testing on all materials in future studies, as we did for three materials in this study. We also recommend having materials reviewed by the intended users.

### Conclusion

Successfully preparing for and responding to disasters requires the close participation of diverse and vulnerable populations. This study adds to the very limited literature available about the availability and readability of emergency preparedness materials from organizations that serve Deaf/HH and older adult clients in a large geographical area, and from a sample of materials drawn from local and national websites. The findings identified an important gap between the estimated health literacy abilities of these two populations and the kind of emergency preparedness materials they can currently access from local CBOs and health departments. Even materials from national websites that had a significant emphasis on emergency preparedness were not adapted to the literacy levels of these focal populations. In our view, a “call to action” is needed to improve practices in creating emergency preparedness materials that are adapted to the needs of these, and other vulnerable populations. Fortunately, there is solid guidance about practical strategies to meet this challenge, especially engaging intended users as co-designers and testers of communication.
